# The Late Dr. H. L. Jones

**Published:** 1915-04-10

**Authors:** 


### Obituary.

THE LATE DR. H. L. JONES.

The death occurred on Sunday last, at the age of fifty-eight, of Dr. Henry Lewis Jones, consulting medical officer to the electrical department at St. Bartholomew's Hospital. Educated at Cambridge and St. Bartholomew's Hospital, he qualified in 1881, and became a Fellow of the Royal College of Physicians in 1894. His main interest was, of course, medical electricity, on which he published a volume which ran into six editions, and made fairly frequent contributions on the subject in the technical press. He was President for 1913-14 of the British Electro-Therapeutic Society, and was also an Associate of the Institution of Electrical Engineers.

